# Ultimate Tensile Strength Evaluation of Bi‐Colored Ethylene‐Vinyl Acetate (EVA) Sheets In‐Office Manufactured With Different Heating Protocols

**DOI:** 10.1111/edt.70036

**Published:** 2025-11-26

**Authors:** Luiz Felipe Rodrigues Siqueira, Ângelo Caetano Rodrigues Mathias Pereira, Bruno Felipe Fernandes, Leon Fernando Marques Jaime, Crisnicaw Veríssimo

**Affiliations:** ^1^ Department of Oral Rehabilitation Federal University of Goiás Goiânia Goiás Brazil

**Keywords:** dental materials, ethylene‐vinyl acetate, mouth protectors, tensile strength, thermoplastic processes

## Abstract

**Background/Aim:**

Bi‐colored mouthguards (MTG) are often sought by both professional and amateur athletes. Bi‐colored MTG can be made in the dental office from premanufactured bi‐colored EVA sheets or custom‐designed in the dental office for greater color variety. This study aimed to evaluate the tensile strength of ethylene‐vinyl acetate (EVA) plates from two commercial brands (BioArt and Polyshok), using different bonding methods and heat sources for producing bicolored MTG.

**Materials and Methods:**

EVA plates from BioArt and Polyshok (3 mm thickness) were bonded using six methods combining two bonding materials (metal angle and glass plate) and three heat sources (heat gun, mini‐torch, and Hannau lamp). Bonded plates were sectioned into 30 standardized bar‐shaped samples (70 × 10 × 3 mm) and subjected to tensile testing. Ultimate tensile strength was recorded, and statistical analyses were performed using One‐Way ANOVA and Tukey's test (α = 0.05).

**Results:**

Significant differences in ultimate tensile strength (MPa) were observed between bonding methods (*p* < 0.001) for each commercial brand. The metal angle and heat gun (MAH) method exhibited the highest tensile strength for both tested EVAs (BioArt: 146.5 ± 30 MPa; Polyshok: 87.8 ± 19.3 MPa). The Hannau lamp produced the lowest values for both tested EVAs (BioArt: 69.8 ± 43.1 MPa; Polyshok: 51.0 ± 14.4 MPa).

**Conclusions:**

The bonding method significantly affects the tensile strength of EVA plates, with the MAH method and heat gun demonstrating superior performance. These findings highlight the importance of optimizing bonding techniques to ensure durability and protective performance in bi‐colored MTG.

## 1 Introduction

Dental‐alveolar traumas result in physical, physiological, aesthetic, and psychosocial consequences, impacting quality of life [1, 2, 3]. It is known that sports activities contribute to an increased risk of injuries and trauma [4, 5]. Accordingly, the use of mouthguards serves as a valuable option for reducing the harmful effects of dental‐alveolar trauma during sports activities [6, 7, 8, 9, 10].

Mouthguards are classified as Prefabricated (Type I), Thermoplastic (Type II), and Custom‐fitted (Type III) [11, 12]. Type III mouthguards are fabricated by the dentist and are the most recommended due to their protective properties for orofacial structures during impact, as well as their comfort, retention, stability, fit, and minimal interference with speech and breathing [13, 14, 15].

For custom‐fitted mouthguards, various materials are employed, including ethylene‐vinyl acetate (EVA), polyvinyl chloride (PVC), natural rubber, soft acrylic, and polyurethane [16, 17]. EVA sheets are the most commonly used due to their impact absorption, stress redistribution, and consequent reduction in stress applied to teeth and supporting tissues [12, 18, 19]. These are available in different commercial brands, easily manufactured, and possess suitable mechanical properties [20].

EVA sheets present distinct mechanical properties related to the elastic modulus (E) and a lack of standardization in copolymer concentrations across brands [21]. Some of these sheets may contain polyurethane, potentially altering their properties as well as the thermoplastic process [22, 23].

Custom‐fitted mouthguards have a significant advantage of customization according to individual patient preferences, achieved by using bi‐colored or tricolored EVA sheets. Only a few commercial brands provide this premanufactured option, requiring dentists to apply various bonding methods, adjusting the heating process and material to create these types of sheets. However, scientific evidence on these bonding methods and their implications for tensile strength is lacking, which can impact the effective fabrication of custom‐fitted mouthguards. Furthermore, prolonged use may lead to delamination in EVA sheets, reducing protective performance, durability, and device quality [24, 25], highlighting the need to better understand these bonding methods.

Thus, this study aimed to evaluate the tensile strength of different commercial brands of EVA (BioArt and Polyshok) bonded using various methods, altering bonding material and heating technique. The null hypothesis formulated for this study is that the bonding methods/heat source will not influence the tensile strength of EVA.

## 2 Material and Methods

**FIGURE 1:**
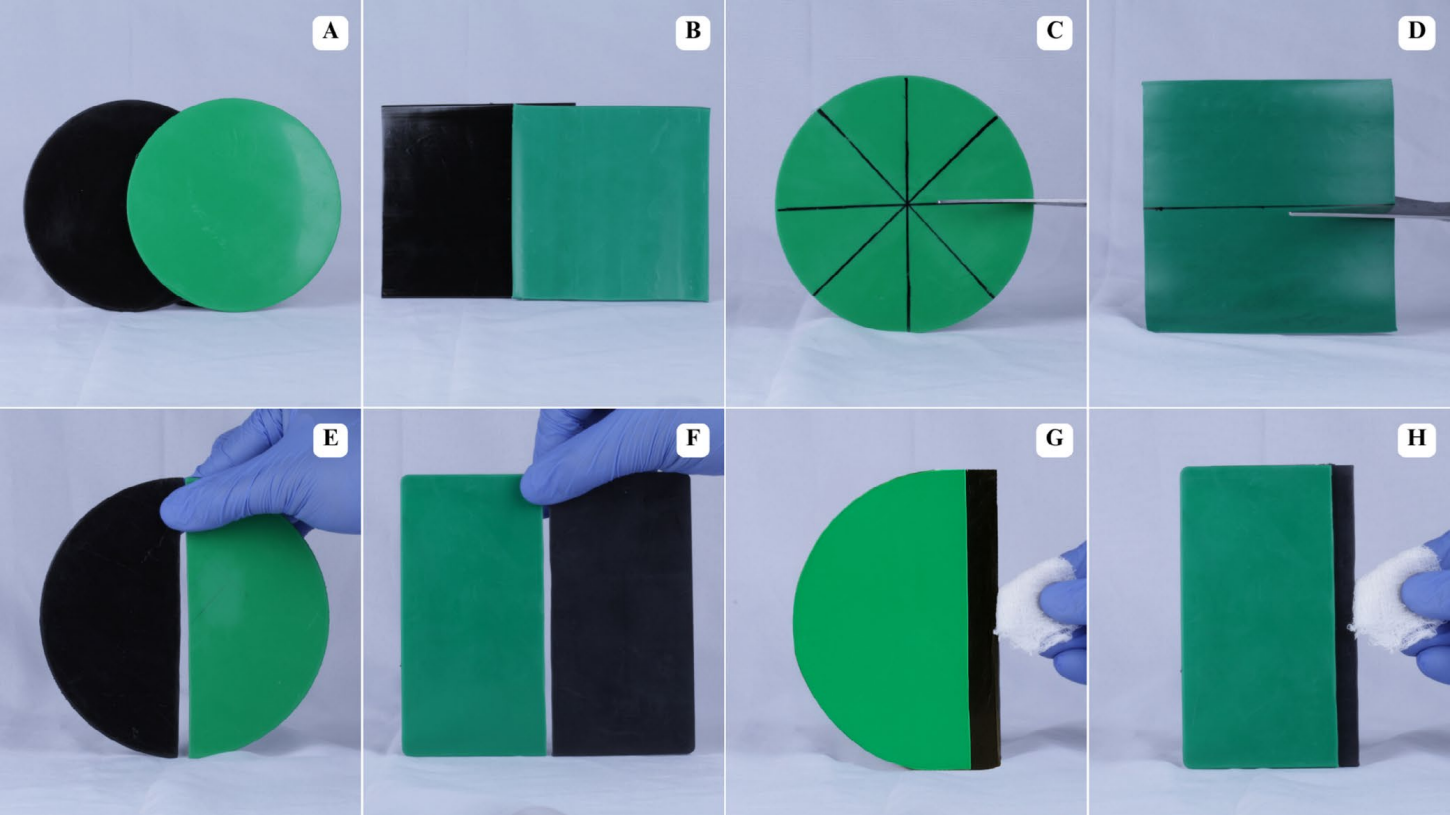
Presentation, sectioning, and cleaning of EVA sheets. (A) BioArt EVA sheets, (B) Polyshok EVA sheets, (C) Delimitation and sectioning of circular sheets, (D) Delimitation and sectioning of square sheets, (E) Final aspect for the fabrication of bicolored circular sheets, (F) Final aspect for the fabrication of bicolored square sheets, (G) Cleaning process of circular sheets, (H) Cleaning process of square sheets.

The EVA sheets used in this study were sourced from BioArt (São Carlos, São Paulo, Brazil) (126 mm diameter) (Figure 1A) and Polyshok (Ohio, Kent, United States) (P) (127 × 127 mm) (Figure 1B), with a 3 mm thickness measured by a digital caliper (Mitutoyo, Aurora, Illinois, United States) and in green and black colors. Sheets were evenly and accurately divided using a millimeter ruler and sharp scissors (Vonder, Curitiba, Paraná, Brazil) (Figure 1C,D), obtaining two semicircles for BioArt with a radius of 63 mm (Figure 1E) and two rectangles for Polyshok (63.5 × 127 mm) (Figure 1F). Sheets were cleaned according to a protocol using acrylic resin monomer (Vipiflash, Pirassununga, São Paulo, Brazil) [26] (Figure 1G,H).

Six bonding methods between the sheets were investigated, varying the surface material type and heating method:

MAH—Metal angle and heat gun;

MAT—Metal angle and mini torch;

MAL—Metal angle and Hannau lamp;

GPH—Glass plate and heat gun;

GPT—Glass plate and mini torch;

GPL—Glass plate and Hannau lamp.

**FIGURE 2:**
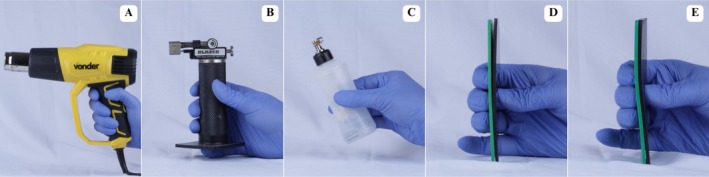
Thermoplasticization of EVA sheets. (A) Heat gun, (B) Mini torch, (C) Hannau lamp, (D) Glossy surface of circular sheets, (E) Glossy surface of square sheets.

**FIGURE 3:**
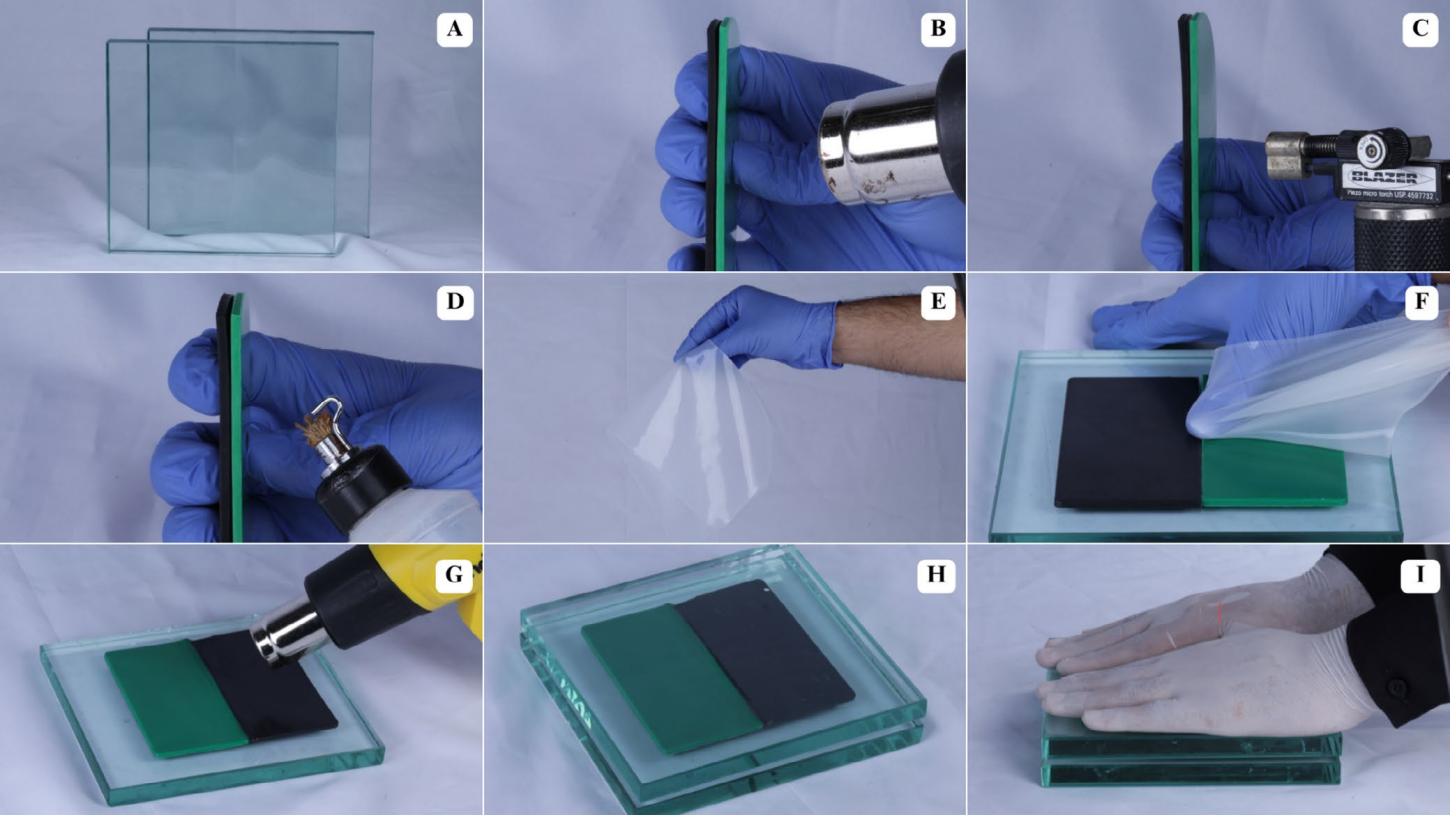
Sheet bonding method using a glass plate. (A) Glass plates used during the bonding method, (B) Heating with a heat gun, (C) Heating with a mini torch, (D) Heating with a Hannau lamp, (E) Selected silicone plastic sheet, (F) Digital pressure using silicone plastic for initial bonding, (G) Additional thermoplasticization, (H) Aspect after bonding and manual pressure positioning, (I) Manual pressure using a glass plate.

**FIGURE 4:**
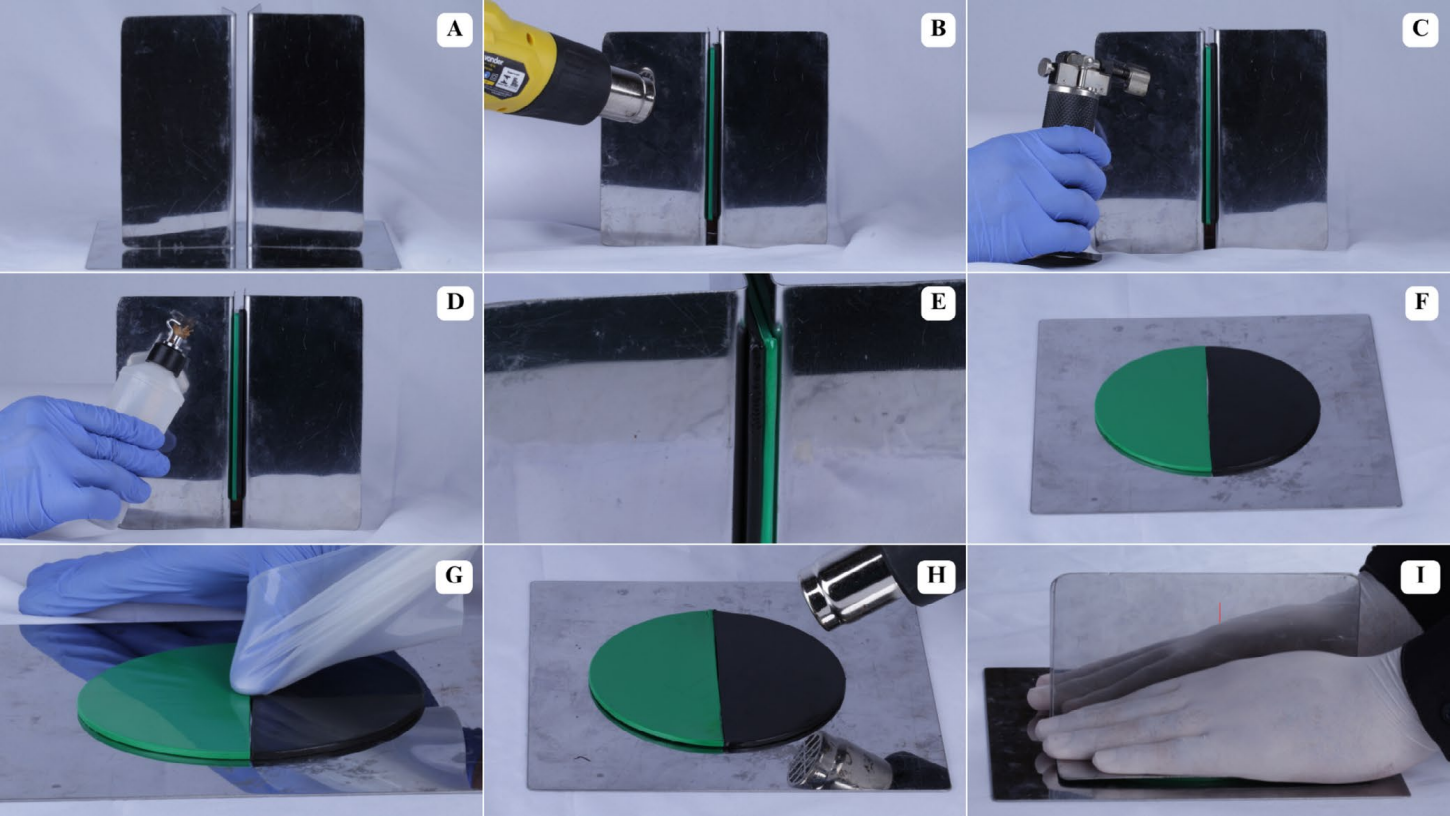
Sheet bonding method using a metal angle. (A) Metal angle and bonding base used for the method, (B) Heating with a heat gun, (C) Heating with a mini torch, (D) Heating with a Hannau lamp, (E) Glossy surface, (F) Aspect after initial bonding, (G) Digital pressure using silicone plastic, (H) Additional thermoplasticization, (I) Manual pressure with metal angle.

Heating was performed at the sheet joint region using a heat gun (Vonder, Curitiba, Paraná, Brazil) (Figure 2A), mini torch (Western, São Paulo, São Paulo, Brazil) (Figure 2B), and Hannau lamp (Konnen, São Paulo, São Paulo, Brazil) (Figure 2C). Total heating time was standardized to 90 s at approximately 80 mm from the EVA sheets, aiming to achieve a glossy appearance on the sheets (Figure 2D,E). Two currently used bonding materials, without scientific validation, were proposed. The first involved thermoplasticizing the joint area, followed by digital bonding and pressing between two glass plates (180 × 180 mm; 1270 g) (Figure 3A), with hand pressure applied by an individual weighing approximately 80 kg (Figure 3B–I). The second involved a proposed device consisting of two metal angles (85 × 165 mm; 165 g) and a metal base (210 × 210 mm) (Figure 4A) [27]. This technique included thermoplasticizing the joint area (Figure 4B–D), followed by digital bonding (Figure 4E–G) and subsequent pressing using metal angles with the same pressure as the previous technique (Figure 4H,I).

**FIGURE 5:**
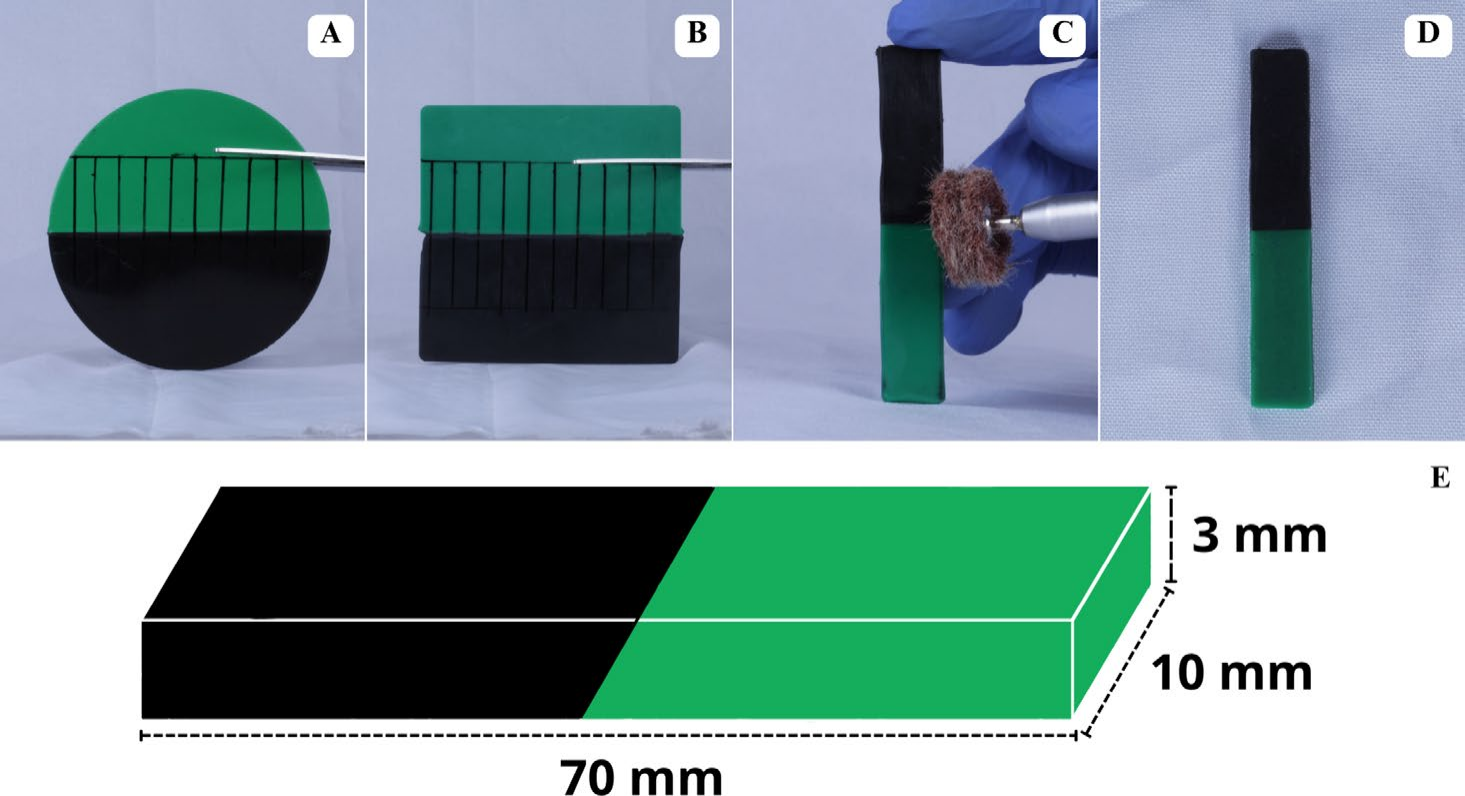
Sample preparation process. (A) Delimitation and cutting of BioArt sheets, (B) Delimitation and cutting of Polyshok sheets, (C) Finishing and polishing of samples, (D) Final aspect, (E) Dimensions established for each sample.

For digital bonding in both techniques, a silicone plastic sheet (36 × 13 cm; 400 g) degreased with 70% alcohol was used to prevent contamination, fingerprints in the joint areas, and burns to the researcher. After sheet bonding, a trained and calibrated operator prepared 30 bar‐shaped samples: 15 from each brand with dimensions of 70 × 10 × 3 mm (Figure 5A–D). Samples were accurately sectioned using sharp straight scissors and measured with a digital caliper [19, 28] (Figure 5E).

**FIGURE 6:**
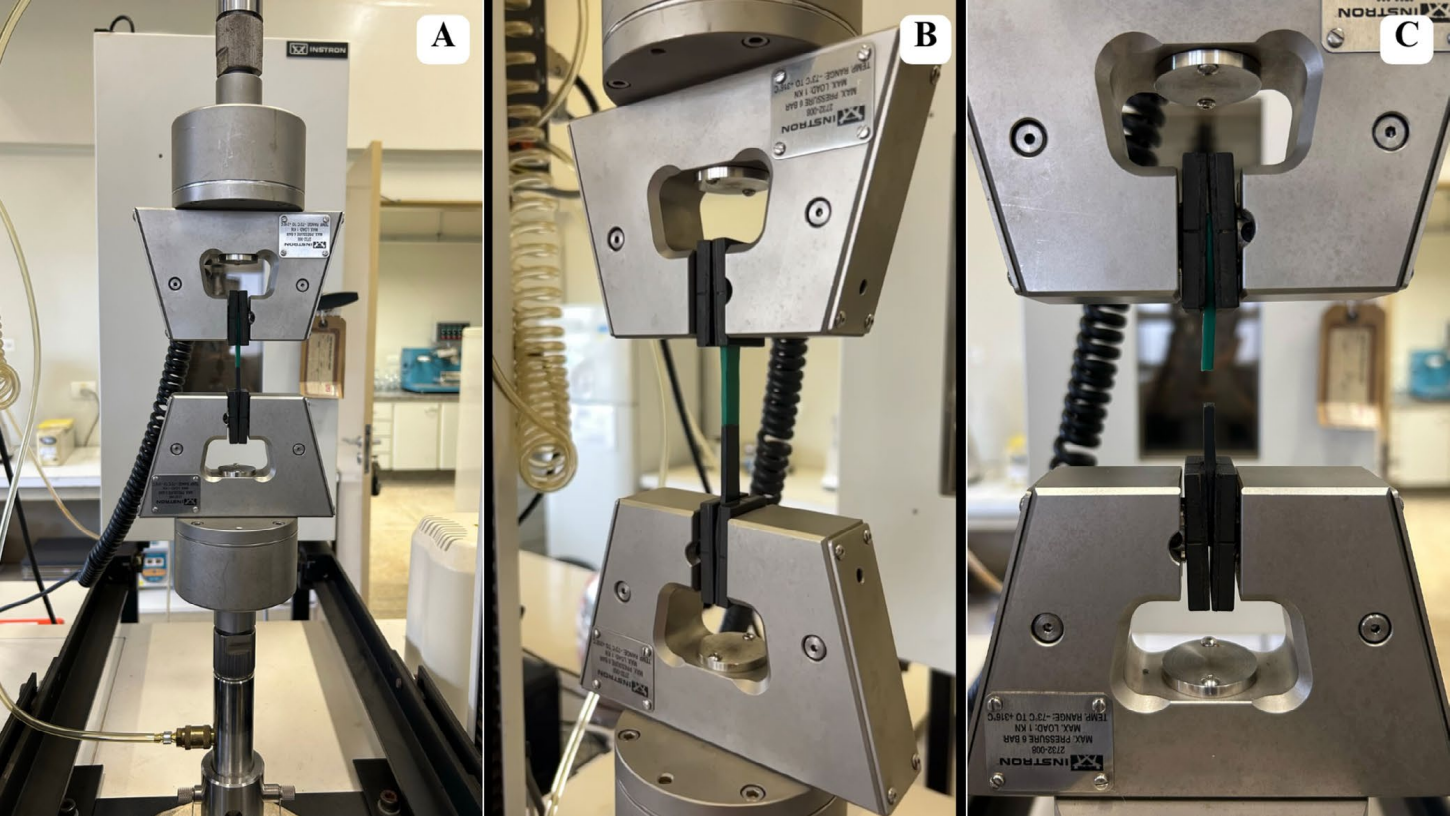
Sample positioned between pneumatic grips for uniaxial tensile testing. (A, B) Application of tensile load on the sample, (C) Rupture moment.

Ultimate tensile strength (MPa) calculations were conducted at the Multiuser Analysis Laboratory, Faculty of Agronomy (LabMulti—UFG). Samples were clamped between two pneumatic grips (2712 Series, Pneumatic Action Grips, Instron Corporation, Norwood, MA, USA) connected to a universal testing machine (Instron, 3367, Grove City, USA) for uniaxial tensile testing as described [19, 28] (Figure 6A). A 5 kN load cell was used, with tensile loading progressively applied from 0 N to the rupture load at a constant speed of 500 mm/min (Figure 6B). During loading, the maximum load was calculated using BlueHill 2 software (Instron Corporation, Norwood, Massachusetts, United States).

After tensile testing, all specimens exhibited adhesive failure modes, characterized by detachment at the bonding interface between the EVA sheets, confirming that rupture occurred in the adhesive zone rather than within the bulk material (Figure 6C).

The statistical analysis of the data indicated a normal distribution using the Shapiro–Wilk test and Brown–Forsythe correction. One‐Way ANOVA test was applied for each commercial brand followed by Tukey's test (α = 0.05). Data was also tested using a Two‐Way ANOVA; however, due to violations in normality tests and equality of variances with Brown–Forsythe correction, it was not possible to perform the test. Therefore, One‐Way ANOVA was chosen to analyze the data for each commercial brand (separately).

## 3 Results

The mean values of ultimate tensile strength, standard deviation, and the results of the Tukey test for Polyshok are shown in Table 1, and for BioArt in Table 2. The bonding method significantly influenced the ultimate tensile strength (MPa) values for the different EVA commercial brands (Polyshok and BioArt), as indicated by the One‐Way ANOVA test (*p* < 0.001).

**TABLE 1:** Mean values (SD) of ultimate resistance (MPa) for Polyshok EVA sheets.

Group	Mean (SD)	Tukey test
MAH	87.8 ± 19.3	A
MAT	74.4 ± 17.4	AB
MAL	56.0 ± 14.8	B
GPL	63.6 ± 16.2	B
GPH	64.0 ± 7.9	B
GPT	51.0 ± 14.4	B

*Note:* Different letters indicate significant differences for Tukey's honestly significant test (*p* < 0.05).

**TABLE 2:** Mean values (SD) of ultimate resistance (MPa) for BioArt EVA sheets.

Group	Mean (SD)	Tukey test
MAH	146.5 ± 30.0	A
MAT	107.3 ± 37.2	B
MAL	69.8 ± 43.1	C
GPL	70.1 ± 38.8	C
GPH	142.3 ± 18.7	AB
GPT	111.4 ± 33.9	AB

*Note:* Different letters indicate significant differences for Tukey's honestly significant test (*p* < 0.05).

For the Polyshok EVA sheets, the MAH (87.8 ± 19.3) and MAT (74.4 ± 17.4) methods showed the highest ultimate tensile strength values (MPa) with no difference between them (*p* = 0.022). No differences were found among the other methods.

For the BioArt EVA sheets, the MAH (146.5 ± 30.0) showed the highest ultimate tensile strength value with no difference for the GPH (142.3 ± 18.7) (*p* = 1.0) and GPT (111.4 ± 33.9) (*p* = 0.071) groups. No difference was found among the MAT, GPH and GPT methods. The lowest values of ultimate tensile strength were found for MAL (69.8 ± 43.1) and GPL (70.1 ± 38.8), and no difference was observed between them (*p* = 1.0).

## 4 Discussion

The null hypothesis (N_0_) that there would be no difference between the bonding methods for EVA plates was rejected. The MAH bonding method and the use of a heat gun showed superiority, while the Hannau lamp demonstrated inferiority in tensile strength for both tested EVAs investigated. For Polyshok, the metal angle bar was the most effective material for bonding, whereas for BioArt, the combination of heating method and bonding material varied significantly between groups, influencing tensile strength.

The fabrication of custom‐fitted mouthguards remains a significant challenge. Ensuring the correct thickness of 3–4 mm, proper design, finishing, and polishing, as well as customization, are crucial factors for producing high‐quality protective devices [19, 29]. Studies show that the use of mouthguards significantly reduces the risk of orofacial trauma in contact sports, improving the protection of dental and dentoalveolar structures [29, 30].

EVA is the predominant material used in mouthguard fabrication due to its favorable balance of mechanical properties and ease of manipulation [13]. However, EVA plates tend to exhibit variability in their manufacturing process, with proprietary compositions often undisclosed, including variations in the copolymer content [21] and possible additions of polyurethane [31]. These factors can directly affect the material's relative stiffness, elasticity, heating time for thermoplastification, and bonding performance.

Understanding the best methods for bonding EVA plates, along with the peculiarities of materials and heat sources used for thermoplastification, is critical. Improper bonding processes can lead to delamination, compromising the structural integrity and protective performance of the mouthguard [30]. All specimens exhibited adhesive failure patterns, characterized by rupture at the bonding interface between the EVA layers. This finding confirms that the tensile rupture occurred at the adhesive zone rather than within the bulk material, highlighting that the differences in mechanical strength among methods were directly related to the quality of the interfacial bonding rather than intrinsic material properties. Nevertheless, from a clinical perspective, whether the failure is adhesive or cohesive EVA becomes less relevant, as any rupture at the interface compromises the integrity and functionality of the mouthguard, potentially rendering it unusable and reducing its protective capacity during sports practice.

A key strength of this study lies in the methodology used for sample preparation, which adhered to previously described protocols to ensure validity and reproducibility [12]. Additionally, the use of high‐speed testing (500 mm/min) to evaluate plate rupture is essential for simulating the impact forces faced by mouthguards during sports activities [28, 30].

The MAH method consistently demonstrated superior performance regardless of the commercial brand, likely due to the high temperature achieved and better heat distribution provided by the metal's high conductivity during bonding. This finding aligns with previous research highlighting the importance of uniform heating for the effective bonding of EVA [30].

The superior performance of Polyshok under the conditions using a metal angle bar may be attributed to its polyurethane content, which likely influences bonding between plates, elastic modulus, and tensile strength [21], combined with the material's excellent thermal conductivity. Additionally, the inclusion of polyurethane enhances elasticity and thermoplastic behavior, both crucial for the efficiency of mouthguards [21]. Conversely, BioArt showed better results with the glass plate, indicating specific material interactions. Pure EVA, being stiffer, may exhibit improved bonding properties with glass. Further studies are needed to elucidate these differences.

The differences between commercial brands and bonding methods are likely related to variations in copolymer concentrations and, consequently, the material's relative stiffness [32, 33]. EVA brands with higher copolymer concentrations tend to be stiffer, as observed with BioArt, which has a higher elastic modulus compared to Polyshok [21]. This increased rigidity modifies the interaction between the material and heat source, as prior studies suggest that the stiffness of pure EVA affects its response to different heating surfaces [13].

The process of bonding EVA plates is critical in the fabrication of custom‐fitted mouthguards, particularly in ensuring effective adhesion between layers for bilamination or multicolor plates. In this context, the performance of heat sources is a determining factor as it directly influences the device's mechanical resistance and durability. Studies indicate that uniform heating improves the alignment of EVA's molecular chains, enhancing bonding strength and reducing the likelihood of structural failures during prolonged use [34, 35].

The heat gun stood out as the most effective heat source, providing consistent results across both brands and bonding materials. Its efficiency in generating uniform heat makes it the preferred method for bonding EVA plates, even when plates of different colors, which may thermoplastify at varying temperatures, are used [36]. Moreover, the heat gun's ability to quickly reach high temperatures optimizes the bonding process, making it a preferred tool in clinical settings.

In contrast, the Hannau lamp showed inferior results, likely due to uneven heat distribution and difficulty in reaching high temperatures. These limitations can lead to inadequate layer adhesion and an increased risk of delamination, compromising the efficacy and safety of the mouthguard. The low thermal variation provided by the Hannau lamp is insufficient to meet the demands of materials like EVA, particularly for commercial brands with specific properties that require uniform heating for effective bonding [37].

The mini‐torch showed intermediate performance, indicating that its application may be a viable, though not ideal, alternative for the bonding process. This result can be explained by the mini‐torch's ability to reach high temperatures relatively quickly, which favors the softening and initial bonding of EVA. However, its effectiveness is limited by the difficulty in ensuring uniform heat distribution across the entire surface of the sheets.

The nature of direct flame heating in the mini‐torch, while time‐efficient, may create overheating points and areas with lower temperatures, leading to variations in the quality of the bonding between the sheets [34, 35]. This lack of uniformity can compromise the alignment of the molecular chains, resulting in lower tensile strength compared to the heat blower, which distributes heat homogeneously [36]. Furthermore, it must be considered that its application requires greater manual control to avoid localized overheating, which may compromise both the integrity of the EVA and the operator's safety.

Exploring advanced technologies, such as lasers or digital heaters, to improve heat uniformity and their impact on EVA adhesion and elasticity appears to be a promising alternative. These innovations could help ensure optimal mouthguard properties after bonding processes, enhancing tear resistance and structural integrity during use, ultimately ensuring athlete safety.

From a clinical standpoint, this study underscores the necessity of employing validated bonding methods and standardized heat sources when manufacturing custom‐fitted mouthguards, as inadequate adhesion between EVA sheets may lead to delamination, tearing, or loss of retention, thereby compromising protective effectiveness. Evidence from lamination studies confirms that bonding strength is strongly dependent on heating temperature [24]. Moreover, investigations into the adhesive strength of laminated mouthguards report that treatment of bonding surfaces (including appropriate heating times and material color) can markedly improve adhesion, directly impacting durability in real‐use scenarios [38]. These findings provide dentists with tangible criteria for selecting bonding protocols that reduce device failure, enhancing both the safety and longevity of mouthguards. This study's limitations include the inability to perform statistical comparisons between EVA commercial brands due to normality and variance testing failures. These restrictions prevent definitive conclusions about which brand performs better but underscore the importance of reporting individual values, providing valuable insights for clinical practice even when comparative analyses are not feasible. Such transparency is essential for clinicians to make informed decisions based on material cost, availability, and performance characteristics.

Furthermore, focusing exclusively on tensile strength as the primary variable may not fully reflect the material's behavior under other conditions, such as impact resistance and long‐term wear. Future studies should address these gaps through more comprehensive evaluations and clinical trials, enabling a deeper and more applicable understanding of the materials used in mouthguard fabrication.

Future studies should guide dentists in selecting bonding methods and fabricating effective, durable, and safe mouthguards capable of withstanding the forces applied during sports activities while providing maximum protection for athletes. Clinical trials on bonded plates after prolonged use and further characterization of EVA are necessary to refine manufacturing techniques and improve material performance.

## 5 Conclusion

- The present study demonstrated that bonding methods and heat sources significantly influence the tensile strength of EVA plates.
- The MAH method, combined with the heat gun, showed superior performance for both BioArt and Polyshok plates, while the use of the Hannau lamp resulted in lower tensile strength values.
- Polyshok achieved better results with the metal angle bar technique, whereas BioArt exhibited variability depending on the bonding method and heat source applied.

## Author Contributions

**Luiz Felipe Rodrigues Siqueira:** conception and design of the study, literature search, experimental development, data acquisition, data analysis and interpretation, manuscript drafting, and manuscript revision. **Ângelo Caetano Rodrigues Mathias Pereira:** experimental development, manuscript preparation, and manuscript editing. **Bruno Felipe Fernandes:** experimental development, manuscript preparation, and manuscript editing. **Leon Fernando Marques Jaime:** literature search, data organization, and manuscript editing. **Crisnicaw Veríssimo:** supervision, conception and design of the study, literature search, critical manuscript revision, and final approval of the version to be published. All authors read and approved the final manuscript.

## Funding

This study was supported by the Brazilian Coordination of Higher Education, Ministry of Education (CAPES) by the PhD scholarship, and Research Support Foundation of the State of Goiás (FAPEG), Brazil (protocol number 202310267000221).

## Conflicts of Interest

The authors declare no conflicts of interest.

## Data Availability

The data that support the findings of this study are available from the corresponding author upon reasonable request.
